# Further Examination of the Performance of Blank Cartridges Used in Captive Bolt Devices for the Pre-Slaughter Stunning of Animals

**DOI:** 10.3390/ani10112146

**Published:** 2020-11-18

**Authors:** Andrew Grist, Randall Bock, Toby G. Knowles, Stephen B. Wotton

**Affiliations:** 1 School of Veterinary Sciences, University of Bristol, Langford House, Langford, North Somerset BS40 5DU, UK; toby.knowles@bristol.ac.uk (T.G.K.); steve.wotton@bristol.ac.uk (S.B.W.); 2 Bock Industries, Inc. (BI), 156 Bock Lane, Philipsburg, PA 16866, USA; randall@bock-industries.com

**Keywords:** animal welfare, blank cartridges, cartridge variation, captive bolt devices, mechanical stunning, performance, stunning, velocity measurement

## Abstract

**Simple Summary:**

Since their advent in 1904, captive bolt devices have been used in abattoirs in Europe to produce an unconscious state in cattle prior to bleeding, to ensure that they suffer no pain during the process. These devices employ a single-use cartridge containing a propellant and no bullet (known as a blank cartridge) to rapidly provide a large quantity of gas through chemical combustion. This gas propels a piston (captive bolt) onto the head of the animal to produce a concussion that is severe enough to produce rapid brain dysfunction, preventing the brain from operating at a state where pain can be perceived by the animal. Subsequent penetration of the bolt is designed to prevent recovery from the stunned state by producing further mechanical damage to the brain. This paper examines and discusses variations in the performance of some blank cartridges in producing sufficient velocity and therefore energy to stun animals, thereby affecting animal welfare at slaughter.

**Abstract:**

Blank cartridges produce gas through deflagration and are used as the main power source in captive bolt devices used within abattoirs and on farms in Europe. The European legislation recognises this and requires users to follow manufacturers’ recommendations in terms of which power cartridge to use. Variation in cartridge performance of Eley (E) cartridges was found in previous research, which was published before Accles and Shelvoke (AS) started full production of their own cartridges. This work examined cartridge performance, structural integrity and dimensional tolerances, and found that the new AS cartridges that varied more greatly in performance in terms of velocity, kinetic energy and mechanical stability of casing than the more established E cartridges. In this study, 15% of the cartridges split at the primer flange on firing, resulting in less kinetic energy, which could impact the ability of the captive bolt to produce a successful stun. This, combined with the variation in performance in a primary component of a device that should have a uniform performance, could lead to animal welfare issues as this variation cannot be predetermined by examination of the cartridge pre firing.

## 1. Introduction

Blank cartridges containing a spun primer (ignition source) and single base propellant (nitrocellulose) within a crimped case are the most common means of kinetic energy generation within captive bolt devices used in Europe for the preslaughter stunning of livestock since 1904 [1,2,3], with pneumatic powered devices being favoured in high-throughput plants, especially in the United States. As an integral component in the successful production of a concussed state, variation in the performance of cartridges that are assumed to be constant in performance has the potential to present a serious welfare concern. Previous work [3] demonstrated a variation in cartridge performance within manufacturers’ batches of cartridges supplied for use in these devices, a variation that could lead to an unsuccessful stun attempt being made due to lack of gas pressure and therefore kinetic energy, with obvious consequences for animal welfare. The previous work found that the kinetic energy output of a cartridge could not be estimated by weighing the cartridge before issuing that cartridge for use. The previous work also discussed anecdotal evidence that issues with mechanical performance of a new manufacturer of cartridges may exist, but corresponding velocity measurements were not undertaken at that time.

This work compares and characterises the performance of two manufacturers’ cartridges, Accles and Shelvoke (AS) (Birmingham, UK) and Eley (E) (Birmingham, UK), both in terms of velocity variability and mechanical properties. For their 4.5 grain (nominal) green coded cartridges (listed as for use on extremely heavy cattle and bulls), the manufacturers, AS, quote an average bolt velocity of 66.7 m/s when measured in air over a distance of 47 mm, 15 mm from the muzzle tip of the tool, and quote a tolerance of ±15% when used in a stunner that is in good condition and maintained in accordance with the instruction manual. The manufacturers also state that this provides a kinetic energy of 544 J [4].

## 2. Materials and Methods

Two hundred cartridges were taken at random from each of a box of 500 Eley ‘’E’’ head stamped, green coded cartridges 0.25” calibre (manufacturer quoted nominal 4.5 grain (0.292 g) propellant). The sample size was selected based on the work involved in processing the cartridges, whilst retaining the aim of being able to differentiate a 1 percent prevalence of faults from a 7 percent prevalence. An exact test with a 0.050 two-sided significance level would have 85% power to detect the difference between a Group 1 proportion of 0.010 and a Group 2 proportion of 0.070 when the sample size in each group was 200.

**Figure 1:**
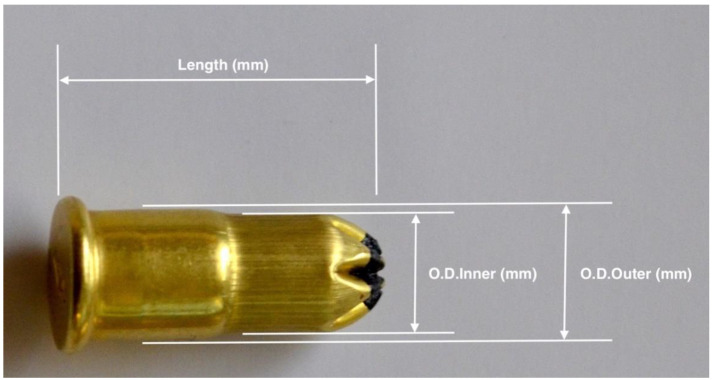
Dimension of cartridges—assessment points recorded for each cartridge.

All the cartridges were within expiry dates and stored in dry conditions. Each cartridge was designated with a number and weighed on a Sartorius balance (Sartorius ENTRIS124-1S Analytical Balance, 120 × 0.0001 g, Sartorius Stedim Biotech North America Inc., New York, NY, USA), and then the figure was recorded. The cartridge dimensions were then measured using Brown and Sharpe, 0 to 200 mm Range, 0.01 mm Resolution, IP67 Electronic Caliper, Stainless Steel with 50 mm Stainless Steel Jaws, 0.03 mm Accuracy callipers (Brown and Sharpe Manufacturing Co., Providence, RI, USA) and the length, outer diameter and inner diameter (Figure 1) were recorded.

The cartridge was then placed within an AS 0.25” Model 5414R “cowpuncher” contact firing penetrating captive bolt device (AS, Birmingham, UK) and fired into a velocimeter that was developed by Bock Industries (Philipsburg, PA, USA) for, and described, in a previous paper [3], with each cartridge being fired at a minimum of 2-min intervals to allow cooling of the device. The velocimeter provides 12 discrete velocity points over the full travel of the penetrating bolt, with a velocity data point every 4 mm for the first 7 zones and then every 8 mm for the next 5 zones [3], and the first bolt velocity measurement being recorded at a point 11.4 mm from the end of the test device.

After firing, the spent cartridge was removed and reweighed to give the mass of propellant fill; it was then cleaned with an acetone swab and reweighed to give a measure of residue. Each cartridge was visually assessed for splits and deformity and split casing at the primer flange.

This procedure was repeated for 200 AS ‘’AS’’ head stamped 0.25” green coded cartridges (manufacturer quoted nominal 4.5 grain) taken at random from a box of 500 cartridges that were stored in the same environment as the Eley cartridges.

All results were entered into a Microsoft Excel (Version 16.5 Microsoft Corporation, Washington, DC, USA) spreadsheet for further analysis, including the following formula for determination of kinetic parameters of the shots.

Kinetic energy was calculated using the formula

E_kin_ = 1/2 m v^2^ (1)

where *m* is the mass of the bolt (kg) and *v* is the bolt velocity (m/s). Using this formula, backward calculation of the manufacturers data sheet has a mass of the bolt of 0.2446 kg. This figure was then used in the kinetic energy range calculations for both sets of cartridges.

### Statistical Analysis

All data were entered into and analysed using the IBM SPSS (v26) statistics package (SPSS Inc., Chicago, IL, USA). Differences in variance were tested using Levene’s test for equality of variances and differences between means tested using a *t*-test assuming equality of means, or not, as appropriate. Differences in proportions as counts were tested by use of exact Chi-square tests.

## 3. Results

### 3.1. Physical Properties

#### 3.1.1. Dimension of Cartridges

**Figure 2:**
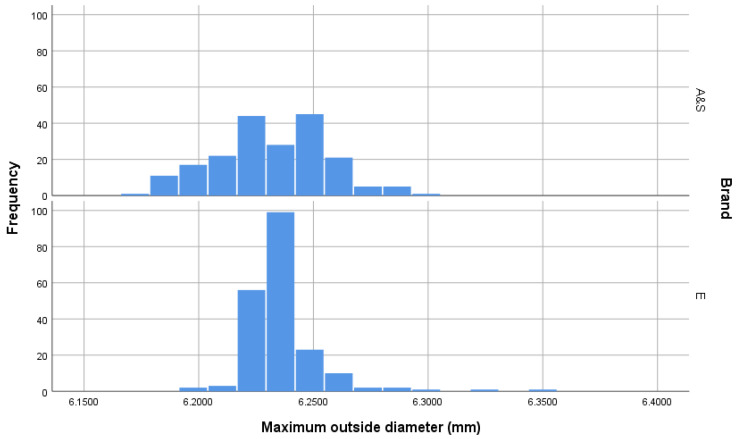
Outer diameter (mm) (OD Outer Figure 1) of cartridges within the sample groups. *n* = 200 per group demonstrating greater tolerance accuracy in the manufacturing process for Eley (E) cartridges as opposed to Accles and Shelvoke (A&S).

**Figure 3:**
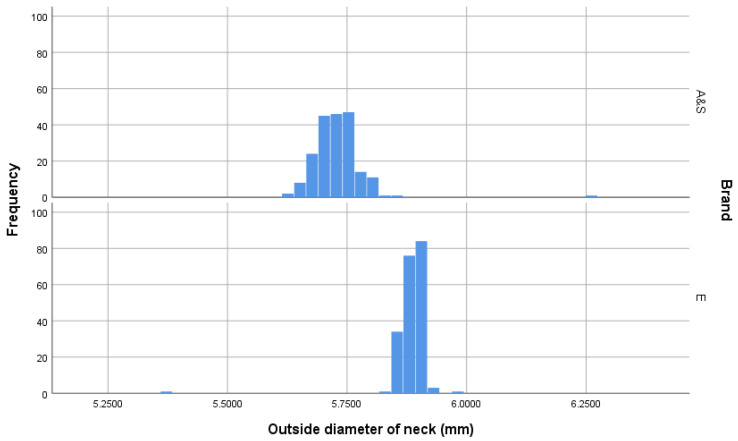
Neck diameter (mm) (OD Inner Figure 1) of cartridges within the sample groups. *n* = 200 per group demonstrating greater tolerance accuracy in the manufacturing process for Eley (E) cartridges and that Accles and Shelvoke (A&S) cartridges had a smaller diameter neck.

**Figure 4:**
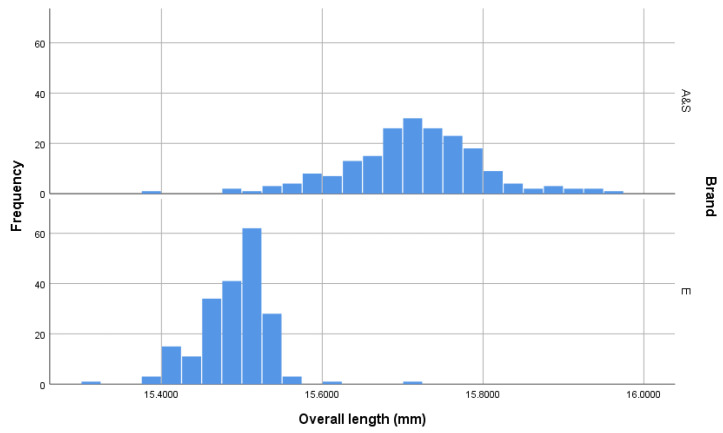
Length of cartridges (mm) within the sample groups. *n* = 200 per group demonstrating greater tolerance in the manufacturing process for Eley (E) cartridges and that the majority of Accles and Shelvoke (A&S) cartridges were longer.

The measurement of cartridge dimensions illustrated that the Eley cartridges had a slightly greater outer diameter (OD Max) (*t* = 2.14, *p* = 0.033) and a lower variation in outer diameter (F = 57.232, *p* < 0.001)(mean and SD; AS = 6.232 mm ± 0.0246 *c.f.* Eley = 6.236 mm ± 0.0169) (Figure 2). Similarly, the Eley cartridges had a slightly greater inner diameter (OD Inner) (*t* = 32.05, *p* < 0.001) with less variation (F = 17.67, *p* < 0.001) (AS = 5.723 mm ± 0.0549 *c.f.* Eley = 5.878 mm ± 0.0405) (Figure 3). The AS cartridges demonstrated a greater mean length (*t* = 303.93, *p* < 0.001) but again more variability (F = 45.02, *p* < 0.001) (AS = 15.717 mm ± 0.0851 *c.f.* Eley = 15.491 mm ± 0.0454) (Figure 4). Ten (5%) of the AS cartridges did not fit into the breach of the test device due to elliptical cross section of the casing, a significantly higher proportion than Eley cartridges, of which all fitted (Chi-sq = 10.26, df = 1, *p* = 0.002).

#### 3.1.2. Splitting at Primer Flange

**Figure 5:**
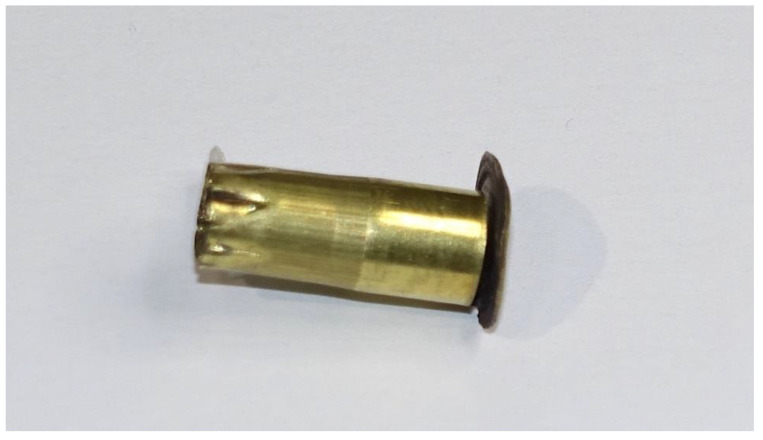
Example of cartridge (AS head stamped) post shot that split at the primer flange.

**Figure 6:**
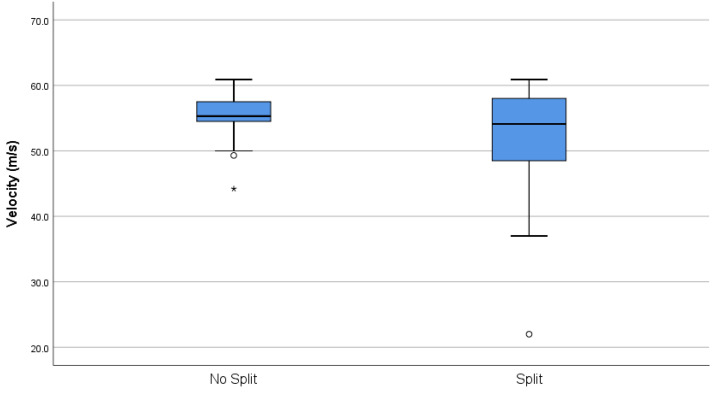
Box plot of velocities recorded at 10 mm extension across both brands where, post firing, a split was found at the primer flange (AS-head-stamped *n* = 30, E-headstamped *n* = 1) or no split was encountered. The dots indicate outliers (quartiles ± 1.5 × the interquartile range), and the asterisks, extreme outliers (quartiles ± 3 x the interquartile range).

**Figure 7:**
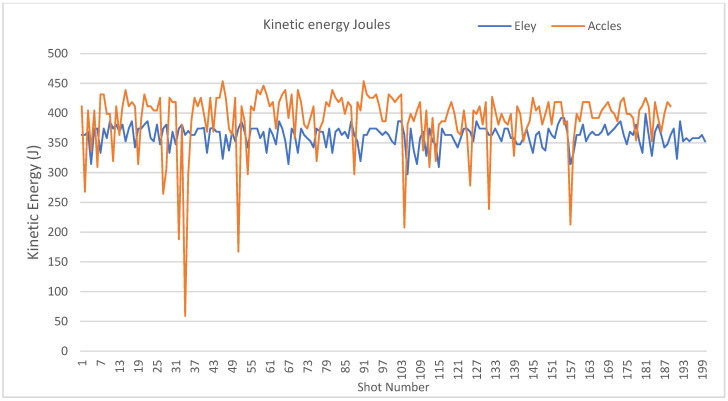
Plot of calculated kinetic energy (Joules) using recorded velocities 11.4 mm from the device. Lower AS head stamp momentum scores corresponding to rupture of the cartridge casing at the primer flange.

On examination post firing, 30 (15%) AS cartridges were found to have split at the primer flange (Figure 5) and 1 (0.5%) Eley cartridge exhibited the same flaw, a significant difference in count (Chi-sq = 29.41, df = 1, *p* < 0.001). Overall, across both brands, these split cartridges produced a lower velocity (*t* = 2.853, *p* = 0.008) with greater variability (F = 135.39, *p* < 0.001) (mean and SD; 51.319 m/s ± 8.4338) than those that did not split at the flange (mean and SD; 56.655 m/s ± 2.235) (Figure 6), and this produced a mean potential kinetic energy range of 224.18 J to 439.56 J in those that split at this point compared with a 329.99 J to 466.18 J range in those that did not split. The variability in kinetic energy can be seen at the individual level in Figure 7, which plots the individual cartridge energies for each brand in sequence of firing. The drops in energy associated with splits in the 30 AS cartridges are clearly visible. The Eley cartridge, which split at the primer flange, was cartridge number 105 and produced a bolt velocity of 49.3 m/s, which was a reduction in comparison to the mean velocity of 54.388 m/s for this head stamp. There was also a difference in mean energy between the two brands, which is apparent in Figure 7 (*t* = 14.631, *p* < 0.001). Although split cartridges are not included in the calculations, the mean energy of the AS cartridges is 400.72 ± SD 29.606 J, and Eley 362.30 ± SD 16.797 J, with significantly greater variation still present within the AS cartridges (F = 21.58, *p* < 0.001) despite the removal of the split cartridges from the calculations.

#### 3.1.3. Case Splitting

**Figure 8:**
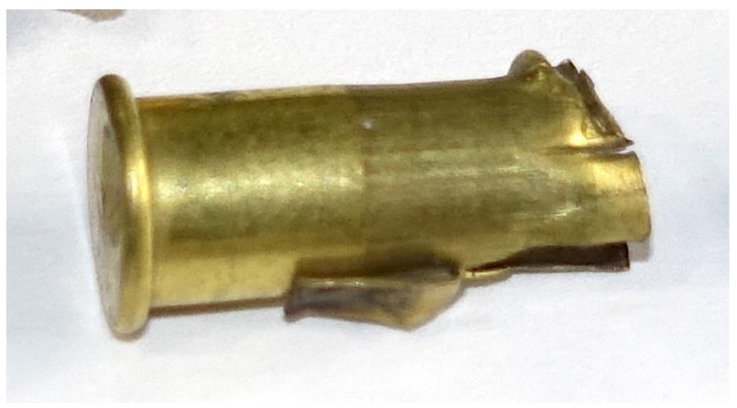
Example of cartridge (AS-head-stamped) post shot that split longitudinally. Note that as in all those that split, the damage terminated at the thicker portion of the case (O.D. Outer Figure 1).

**Figure 9:**
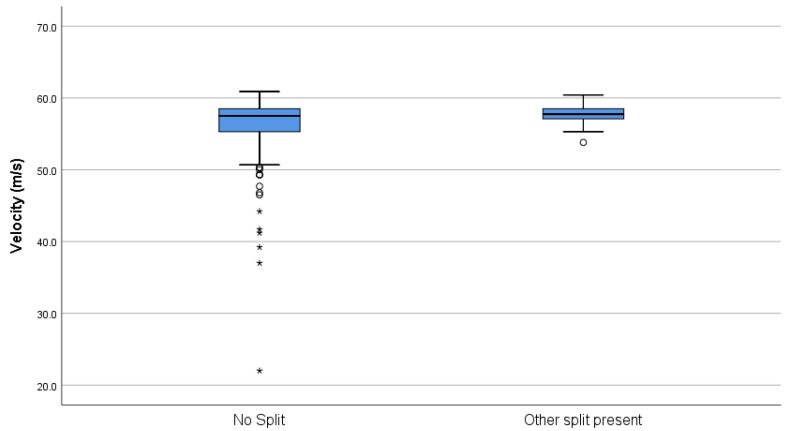
Plot of velocities recorded at 11.4 mm extension for AS cartridges where, post firing, a longitudinal split *n* = 40 (labelled “other split’’ to differentiate from primer flange split) was found in the case or no split was encountered. The dots indicate outliers (quartiles ± 1.5 × the interquartile range), and the asterisks, extreme outliers (quartiles ± 3 × the interquartile range).

On examination post firing, 40 (20%) AS cartridges were found to have a longitudinal split in the case (Figure 8); no Eley cartridges demonstrated this fault, a significant difference between brands (Chi-sq = 44.44, df = 1, *p* < 0.001). However, the results demonstrated that cartridges that developed a longitudinal split of the case had a greater propellant fill weight (Figure 9) than other AS cartridges (*t* = 3.40, *p* = 0.001) (5.780 ± 0.4630 g *c.f.* 5.524 ± 0.2886 g) and produced a higher velocity (*t* = 3.95, *p* < 0.001) (57.695 ± 1.3351) m/s) than those AS cartridges that did not split (55.904 ± 4.9084 m/s) (Figure 9).

### 3.2. Weight of Cartridges and Propellant Load

**Figure 10:**
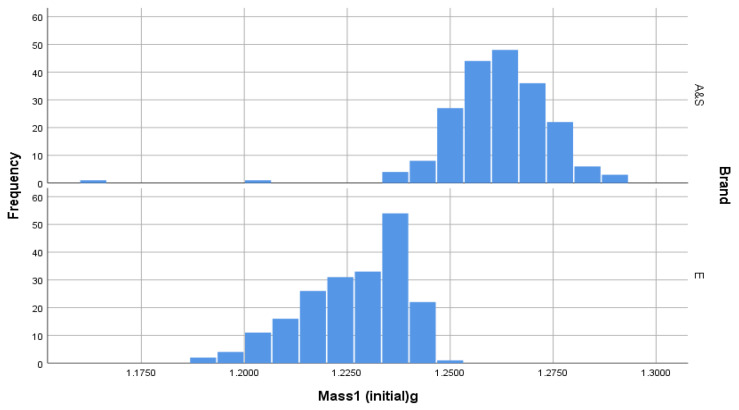
Initial mass of unfired cartridges illustrating that AS-head-stamped cartridges were heavier than E-head-stamped cartridges.

Initial weighing of cartridges showed that AS-head-stamped cartridges were heavier than E-head-stamped cartridges but that the distributions of the weights of each brand were not significantly different (Table 1 and Figure 10).

**Figure 11:**
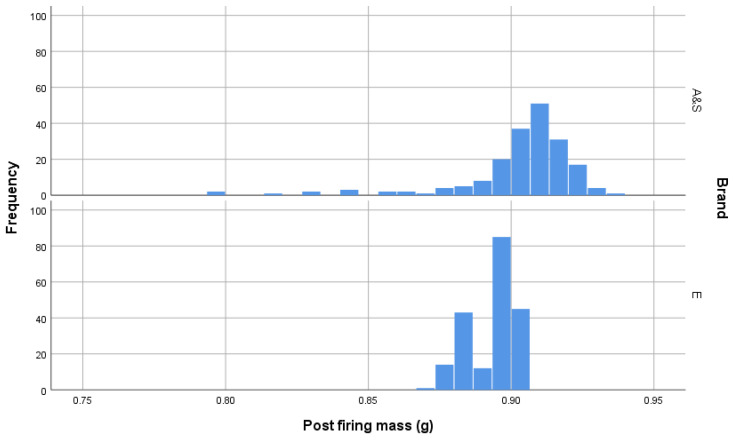
Plot of cartridge weights in grammes post firing.

Post firing weighing of cartridges showed that AS-head-stamped cartridges were heavier than E-headstamped cartridges and that the distributions of the weights post firing of each brand significantly differed (Table 1 and Figure 11).

**Figure 12:**
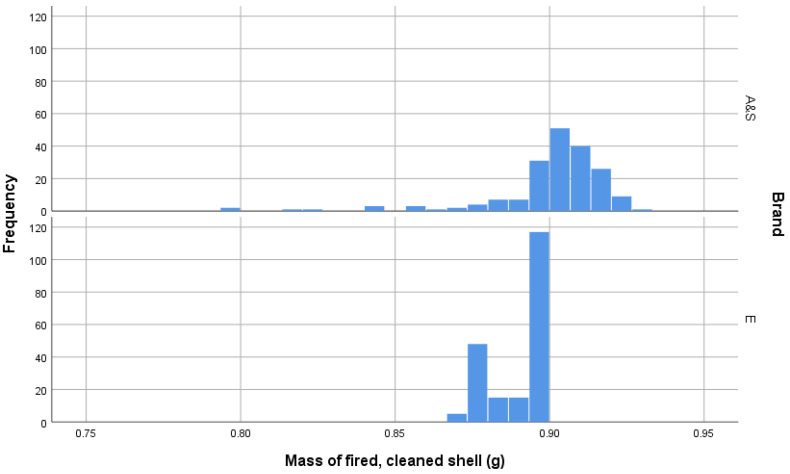
Plot of cartridge weights post firing after residue has been removed by cleaning.

Once cleaned of residue, weighing of cartridges showed that AS-head-stamped cartridges were heavier than E-headstamped cartridges and that the distributions of the cleaned cartridge weights of each brand significantly differed (Table 1 and Figure 12).

### 3.3. Post Firing—Propellant Fill

**Figure 13:**
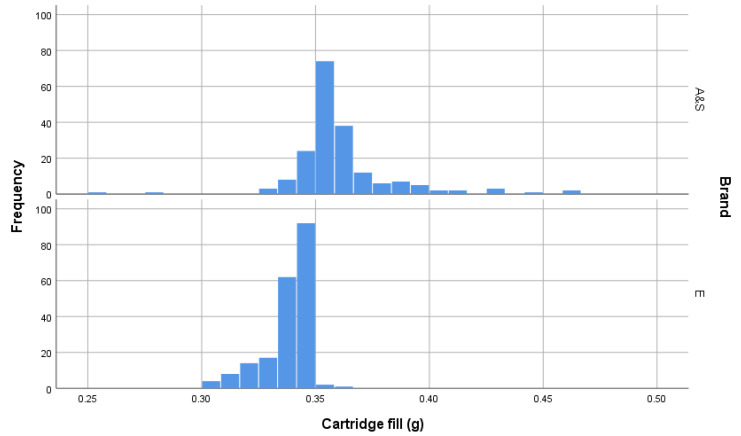
Plot of propellant fill in grammes, calculated by subtracting the mass of the post firing and cleaned cartridge cases from the pre-firing mass.

Calculated by subtraction, the fill weight of cartridges showed that AS-head-stamped cartridges had greater mean fill than the E-head-stamped cartridges and that the distributions of the fill weights, post firing, of each brand differed significantly (Table 1 and Figure 13). E-head-stamped cartridges (*n* = 200) had a mean propellant fill of 0.3378 g (±0.01019) and the AS (*n* = 190)-head-stamped cartridges had a mean propellant fill of 0.3612 g (±0.02260) (Figure 13). The nominal grain for both of these cartridges was quoted as 4.5 grain; both types of cartridge contained more propellant, with E-head-stamped containing 5.21 grain mean and AS-head-stamped containing 5.57 grain mean (1 grain = 0.065 g).

### 3.4. Performance Properties

#### Bolt Velocity Measurements

**Figure 14:**
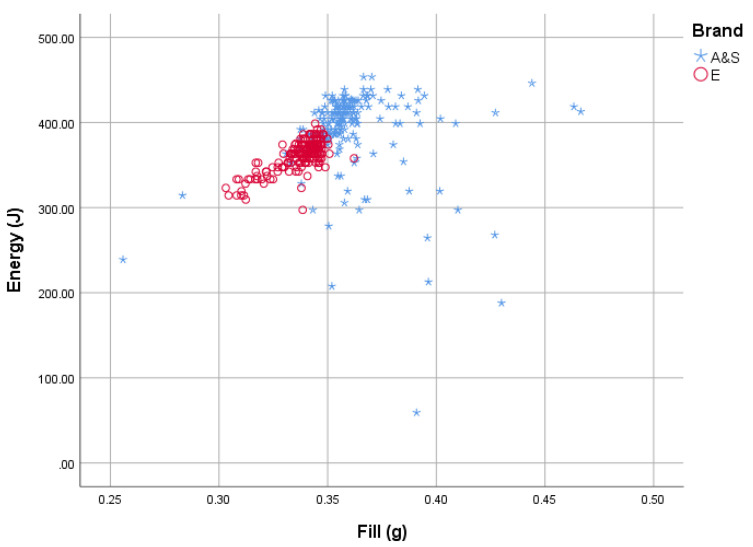
Scattergraph of calculated kinetic energy (J) against propellant fill weight (g).

E-head-stamped cartridges (*n* = 200) produced a mean bolt velocity of 54.388 m/s (±1.3233), and the AS-head-stamped cartridges (*n* = 190) produced a mean bolt velocity of 55.985 m/s (±5.9529) (Figure 13). Propellant fill did have an effect on velocity recorded and hence calculated kinetic energy; however, as can be seen in Figure 14, the AS-head-stamped cartridges displayed a less uniform proportional increase than the E-head-stamped cartridges. Note that as the bolt mass is a constant, velocity and kinetic energy are directly related.

## 4. Discussion

European legislation and United Kingdom legislation [5,6,7,8] require abattoirs, as regards stunning, to take into account the manufacturers’ recommendations (Article 6, 2(a)), and that the correct strength of cartridge or other propellant is used, in accordance with the manufacturer’s instructions, to produce an effective stun [6,7,8,9]. This requirement recognises the importance of the cartridge choice, but also infers an expectation of unform performance of the cartridges. As detailed in the introduction, the manufacturer data sheet for these cartridges quotes an average velocity of 66.7 m/s and a kinetic energy of 544 J. Given the manufacturers’ quoted performance tolerances of 15% for velocity and kinetic energy, the cartridges should produce a velocity within the range of 56.70–73.37 m/s. However, this figure of 15% variation for both parameters is slightly misleading; given that kinetic energy (*E_kin_*) is 1/2 mv^2^ (where *m* is the mass of the bolt and *v* is velocity), a 15% variation in velocity has the potential to produce a larger than 15% variation in kinetic energy within a range of 393 J to 658 J.

### 4.1. Cartridge Dimensions

The Eley cartridges appear to have been produced to a stricter tolerance range than the AS cartridges. The measurement of cartridge dimensions illustrated that the Eley cartridges had a lower variation in outer diameter (OD Max) (AS = 6.232 mm ± 0.0246 *c.f.* Eley = 6.236 mm ± 0.0169) (Figure 2), inner diameter (OD Inner) (AS = 5.723 mm ± 0.0549 *c.f.* Eley = 5.878 mm ± 0.0405) (Figure 3) and length, with the AS cartridges demonstrating a longer mean length (AS = 15.717 mm ± 0.0851 *c.f.* Eley = 15.491 mm ± 0.0454) (Figure 4).

### 4.2. Cartridge Splitting

Two types of splitting of AS cartridge cases were observed post shot, either a longitudinal split (20% of the cartridges) along the length of the thinner section of the body (OD Min) terminating at the thicker portion (OD Max) (Figure 1 and Figure 7), or a separation between the body and rim containing the primer (15% of the cartridges) (Figure 5). In terms of performance, the cases with a longitudinal split of the case were found to be associated with a higher propellant fill and demonstrated a subsequent higher velocity. In terms of animal welfare, this higher velocity output is beneficial; however, the removal of the spent cartridge from the device was difficult and took time due to the split allowing expansion of the cartridge within the breech. This could lead to an operative potentially damaging the breech in an effort to remove as much as 20% of cartridges from the device post shot.

Those cartridges that split between the body and rim containing the primer, the primer flange, (15% AS-head-stamped cartridges) produced less velocity, which correlates with the observed exhaust gas emanating from the cap of the device when this rupture of the casing occurred, and also produced significantly less kinetic energy at a point 11.4 mm from the device. In these cases, the split allowed propellant gas to escape rearward rather than propelling the captive bolt forward. This flange split occurrence bore no relation to fill weight and possibly represents a manufacturing issue. This velocity decrease could be an issue in terms of stunning potential, and therefore animal welfare at slaughter, as the calculated kinetic energies for these cartridges were markedly lowered. There appeared to be no pre-firing indicators as to the likelihood of this occurrence.

### 4.3. Cartridge Fill

Both sets of cartridges had a higher propellant fill than the quoted nominal 4.5 grains (0.292 g). As cartridges are usually filled volumetrically this can occur, and in terms of stunning, the higher propellant fill should lead to a corresponding increase in velocity and thus kinetic energy, improving the chances of a successful stun on the first attempt.

### 4.4. Cartridge Performance

Generally, the AS cartridges produced a higher velocity than those of Eley but had a larger range in their performance both in terms of velocity and mechanical structural stability, with 35% (*n* = 71) of cartridges displaying damage post shot (AS *n* = 70/200, E *n* = 1/200). Neither cartridge reached the manufacturers quoted speed of 66 m/s.

## 5. Conclusions

Stunning is the main method of penetrating captive bolt propulsion used in Europe, and the operators that stun animals expect the blank cartridge they use to perform in a uniform and predictable manner. This work demonstrates that some commercially available cartridges have a variation in performance within a batch that could pose an issue for successfully stunning an animal on the first attempt, a requirement of European legislation. Anecdotally, it has also led to the operators having a lowered confidence in their equipment performing as expected.

## 6. Patents

The Bock Industries Velocimeter will be patented, and as such the setup and methodology is not described in detail in this paper.

## Figures and Tables

**Table 1:** Mean, SD and the results of the Levine’s test and *t*-test comparing the cartridge weight components between the two brands of cartridge.

	Eley		AS		Levine’s Test		*t*-Test	
	Mean	SD	Mean	SD	F	*p*	*t*	*p*
Initial Mass (g)	1.227	0.0125	1.262	0.0131	3.53	0.063	27.24	<0.001
Mass Post Firing (g)	0.893	0.0084	0.903	0.0210	22.71	<0.001	5.71	<0.001
Mass Cleaned Shell (g)	0.889	0.0081	0.901	0.0199	17.86	<0.001	7.45	<0.001
Fill Mass (g)	0.338	0.0102	0.361	0.0226	20.6	<0.001	13.04	<0.001
